# Supplementary material for: Freedom to choose between public resources promotes cooperation
Source: PLoS Comput Biol. 2021 Feb 8;17(2):e1008703. doi: 10.1371/journal.pcbi.1008703 (PMC7895419; doi:10.1371/journal.pcbi.1008703)
Supplement: S1 Text — Overview and further analysis of the model, discussion of the dependence on the parameters, and presentation of computer codes. (PDF) [file pcbi.1008703.s001.pdf]

Supporting Information S1 Text

Freedom to Choose between Public Resources Promotes Cooperation

Mohammad Salahshour

A. OVERVIEW OF THE MODEL

In the general case, we consider a population of N individuals who can play one of n possible public goods games (PGGs). Each individual has a preferred game i, which can be one of the n possible PGGs, and a strategy x, which can be cooperation (C), or defection (D). At each time step, groups of g individuals are drawn at random from the population pool. Individuals in each group enter their preferred PGG and play the game. That is, in each PGG, cooperators pay a cost c to invest an amount c to the public resource, and defectors pay no cost and do not invest. All the investments are multiplied by an enhancement factor (r_i for PGG i) and are divided equally among all the individuals who participated in that PGG. In addition to the payoff received from playing the PGG, we assume individuals gather a base payoff π_0 from other activities not related to the PGG. After receiving the payoffs, a selection occurs in which individuals reproduce according to their payoff. In the selection stage, the whole population is updated such that the population size remains constant. More precisely, each individual in the next generation is offspring to an individual in the past generation with a probability proportional to its payoff. Offspring inherit the strategy and the preferred game of their parent, subject to mutations. Mutations in the preferred game and the strategy of the individuals happen independently, each with probability ν. If a mutation in the preferred game occurs, the preferred game of the offspring is set equal to a randomly chosen PGG, other than that of its parent. In the same way, if a mutation in the strategy of an individual occurs, its strategy is set equal to a strategy other than the strategy of its parent (C to D and vice versa).

B. ANALYSIS OF THE MODEL WITH $n = 2$ PUBLIC RESOURCES

To perform a detailed analysis of the model with $n = 2$, we set $g = 10$, $\nu = 10^{-3}$, $c = \pi_0 = 1$, and present the results of numerical solutions of the replicator dynamics in Fig. (A). The results of a simulation in a population of size $N = 20000$, for the same parameter values, is presented in Fig. (B.a) to Fig. (B.f). In Fig. (A.a) and Fig. (A.b), we present contour plots of, respectively, the time average cooperation level in game 1, $\langle \rho_C^1 \rangle_t$, and the time average cooperation level in game 2, $\langle \rho_C^2 \rangle_t$. Here, and in the following, $\langle . \rangle_t$ denotes a time average. In Fig. (A.c), the contour plot of the time average density of the individuals in game 1, $\langle \rho^1 \rangle_t = \langle \rho_C^1 + \rho_D^1 \rangle_t$ is presented. Due to the symmetry of the two games, the time average density of the individuals in game 2 is similar, and results from the reflection of Fig. (A.c) with respect to the diagonal. Here, the initial condition is a homogeneous initial condition in which the initial density of all the strategies equals 0.25 (that is $\rho_x^i = 0.25$ for $i = 1$ and 2, and $x = C$ and D).

As can be seen in Fig. (A.a) and Fig. (A.b), the density of the cooperators is higher in the game with the higher enhancement factor. This makes the game with higher enhancement factor, on average, the more profitable one as well. As a result, as can be seen in Fig. (A.c), the density of individuals is higher in the game with the higher enhancement factor. Comparison with the results of simulations in Fig. (B.a) and Fig. (B.b), where respectively, the time average density of cooperators in game 1, and the density of individuals who prefer game 1, are plotted, shows the same is true for the result of simulations in finite populations. Here, as the initial condition of the simulations, strategies and the preferred games of the individuals are assigned uniformly at random (this is equivalent to a homogeneous initial condition used for the solutions of the replicator dynamics). Comparison of the results of the simulation with the solutions of the replicator dynamics shows a high agreement between the two.

To see in what parameter region the dynamic settles into a fixed point or it shows cyclic behavior, we consider the standard deviation of the time series of the cooperation level in the population, $\sigma_{\rho_C} = \langle (\rho_C - \langle \rho_C \rangle_t)^2 \rangle_t$. When the dynamics settle in a fixed point, this quantity equals zero. On the other hand, when the attractor of the dynamics is a periodic orbit, σ_{ρ_C} takes a non-zero value. Thus, σ_{ρ_C} distinguishes between different attractors of the system. σ_{ρ_C} is plotted in Fig. (A.d) for the solutions of the replicator dynamics, and in Fig. (B.d), for a simulation with the same parameter values. Again, comparison reveals a high level of agreement between the result of simulations

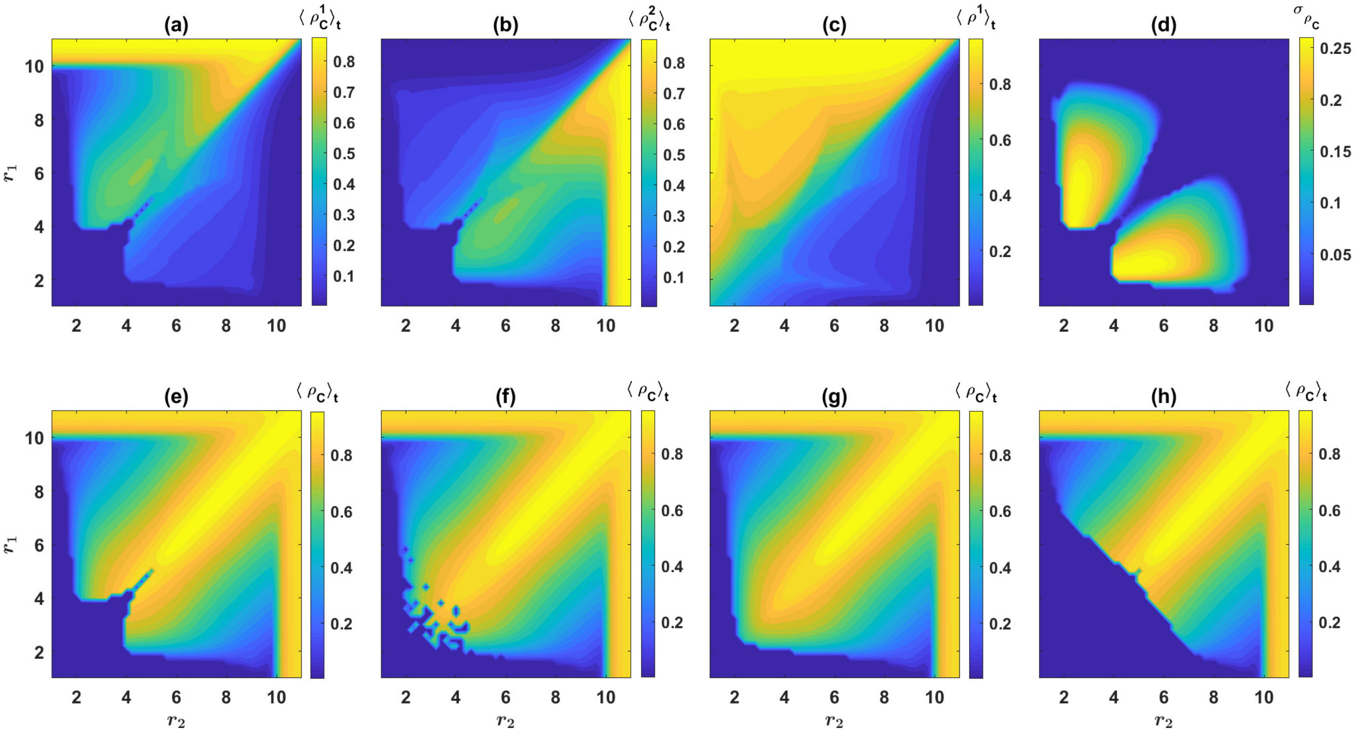

FIG. A. Numerical solution of the replicator dynamics. (a) to (c): (a) and (b) present color plots of respectively $\langle \rho_C^1 \rangle_t$ and $\langle \rho_C^2 \rangle_t$, and (c) is the color plot of the time average density of individuals in game 1, $\langle \rho^1 \rangle_t = \langle \rho_C^1 + \rho_D^1 \rangle_t$. It can be seen that the density of individuals and cooperators is the highest in the higher quality resource. (d): The standard deviation of the density of cooperators, which can be used as a measure of the strength of cyclic behavior. (e) to (h): The time average density of cooperators starting from different initial conditions. In (e) the initial condition is homogeneous ($\rho_C^1 = \rho_C^2 = \rho_D^1 = \rho_D^2 = 0.25$), in (f), it is a randomly chosen initial condition in which $\rho_x^j = \frac{a_x^i}{a_C^1 + a_C^2 + a_D^1 + a_D^2}$, where, a_x^j for $i = 1$ and 2, and $x = C$ and D, are random numbers chosen uniformly at random in the interval [0,1]. In (g), the initial condition is a cooperation favoring one ($\rho_C^1 = \rho_C^2 = \rho_D^2 = 0$ and $\rho_D^1 = 1$), and in (h) the initial condition is a defection favoring one ($\rho_C^1 = \rho_C^2 = 0$ and $\rho_D^2 = \rho_D^1 = 0.5$). Here, $g = 10$, $\nu = 10^{-3}$, and $c = \pi_0 = 1$. The replicator equations are solved for $T = 4000$ time steps and the time averages (or standard deviation) are calculated based on the last 2000 time steps. In (a) to (d), the initial condition is a homogeneous initial condition.

and the solutions of the replicator dynamics. However, we note that in the case of simulations in finite populations, due to population stochasticities, the standard deviation of the cooperation level deviates slightly from zero even in the region where the replicator dynamics settle into a fixed point. In passing, we note that, as can be seen by the sudden change in the value of σ_{ρ_C}, while the transition from the defective fixed point to the periodic orbit in small enhancement factors shows bistability and is discontinuous, the transition from the cyclic behavior to the cooperative fixed point in large enhancement factors shows no bistability and is gradual. That is, in large enhancement factors, by increasing the enhancement factors, the extent of fluctuations as measured by σ_{ρ_C} decreases continuously, until it reaches zero at the bifurcation line, and the dynamics settle into a fixed point.

As we have seen in the main text, for small enhancement factors the dynamics show bistability and its stationary state depends on the initial condition. To see how this happens, we solve the replicator dynamics with different initial conditions and present the resulting time average cooperation level $\langle \rho_C \rangle_t = \langle \rho_C^1 + \rho_C^2 \rangle_t$, in Fig. (A.e) to Fig. (A.h). In Fig. (A.e), the initial condition is a homogeneous initial condition. That is, the initial density of all the four strategies are the same, and equal 0.25 ($\rho_C^1 = \rho_C^2 = \rho_D^1 = \rho_D^2 = 0.25$). The phase boundary between the defective fixed point and the periodic orbit can be determined based on the final state of the dynamics, starting from this initial condition. To see how the system behaves starting from different initial conditions, in Fig. (A.f), we present the contour plot of the time average cooperation level starting from different, randomly chosen initial conditions. Here, the $r_1 - r_2$ plane is divided into small cells, and for each cell a random initial condition is used to solve the replicator dynamics. The choice of the initial condition is such that, for each cell we set $\rho_x^i = \frac{a_x^i}{a_C^1 + a_C^2 + a_D^1 + a_D^2}$, where a_x^is for $i = 1$ and 2, and $x = C$ and D, are random numbers chosen uniformly at random in the interval [0,1]. As can be seen by comparison of Fig. (A.e) and Fig. (A.f), for both very large and very small enhancement factors, the

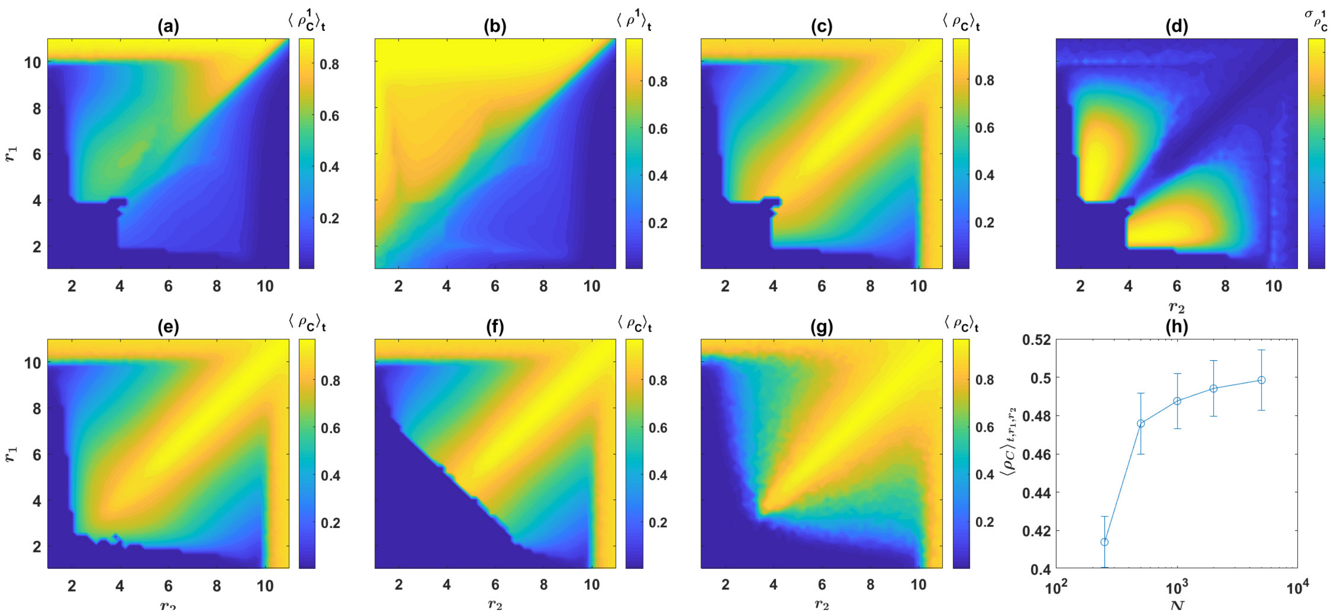

FIG. B. Simulations of the model. (a) and (b): (a) presents the color plot of $\langle \rho_C^1 \rangle_t$ and (b) is the color plot of the time average density of individuals in game 1, $\langle \rho^1 \rangle_t = \langle \rho_C^1 + \rho_D^1 \rangle_t$. It can be seen that the density of individuals and cooperators is the highest in the higher quality resource. (c) and (d): (c) presents the time average density of cooperators starting from a homogeneous initial condition ($\rho_C^1 = \rho_C^2 = \rho_D^1 = \rho_D^2 = 0.25$), and (d) presents its standard deviation, which can be used as a measure of the strength of cyclic behavior. (e) and (f): The time average density of cooperators starting from a cooperation favoring initial condition ($\rho_C^1 = \rho_C^2 = \rho_D^2 = 0$ and $\rho_D^1 = 1$) (e), and the time average density of cooperators starting from a defection favoring initial condition ($\rho_C^1 = \rho_C^2 = 0$ and $\rho_D^2 = \rho_D^1 = 0.5$) (f). (g): The density of cooperators starting from a homogeneous initial condition in a population of size $N = 500$. Comparison to (c) for $N = 20000$ shows transition to the cyclic phase occurs for smaller enhancement factors for larger population sizes. (h): The mean of the time average density of cooperators over the phase diagram ($q \geq r_1, r_2 \leq g$) as a function of population size. It can be seen that the level of cooperation increases with increasing the population size. Here, $g = 10$, and $\nu = 10^{-3}$. In (a) to (d), and (f) $N = 20000$, and in (e) $N = 10000$, and the simulations are performed for $T = 5000$ time steps. In (g) $N = 500$ and the simulation is performed for $T = 25000$ steps. The averages are taken after discarding the first 500 time steps.

stationary state of the dynamics does not depend on the initial condition. However, for medium enhancement factors, two different stationary states, a defective fixed point in which cooperation does not evolve, and a periodic orbit in which cooperation evolves, are possible. Starting from a randomly chosen initial condition, the dynamics settle in one of these two attractors.

Analysis of the model reveals the most cooperation favoring initial condition is an initial condition in which all the individuals prefer the same game. This can be seen by noting that, starting from such an initial condition, mutant cooperators who prefer the unoccupied game receive the highest payoff and grow in number. When the density of such cooperators increases enough, mutant defector who prefer the same game obtain the highest payoff and thus the highest growth. At this stage, the cyclic dominance of cooperators and defectors in the two games sets in. The time average cooperation level starting from one such cooperation favoring initial condition is plotted in Fig. (A.g). Here, the initial condition is $\rho_C^1 = \rho_C^2 = \rho_D^2 = 0$ and $\rho_D^1 = 1$. As mentioned before, any initial condition in which all the individuals prefer the same game is equally cooperation favoring and would result in the same picture. The lower boundary of the phase diagram, that is the line above which the periodic orbit becomes stable, can be determined starting from such an initial condition. On the other hand, the most defection favoring initial condition is the one in which all the individuals are defectors and all the different games are occupied by the same number of individuals. The time average cooperation level in the stationary state of the dynamics starting from such an initial condition is plotted in Fig. (A.h). This determines the upper boundary of the coexistence region above which the defective fixed point becomes unstable.

To compare the solutions of the replicator dynamics, with the results of simulations, in Fig. (B.c), Fig. (B.e), and Fig. (B.f), we plot the time average cooperation level, resulted from a simulation in a population of size $N = 20000$, starting from different initial conditions. In Fig. (B.c), the initial condition is a homogeneous initial condition, in which the strategy and the preferred games of the individuals are assigned uniformly at random, in Fig. (B.e), the initial condition is a cooperation favoring one. As mentioned before, this can be any initial condition in which all the

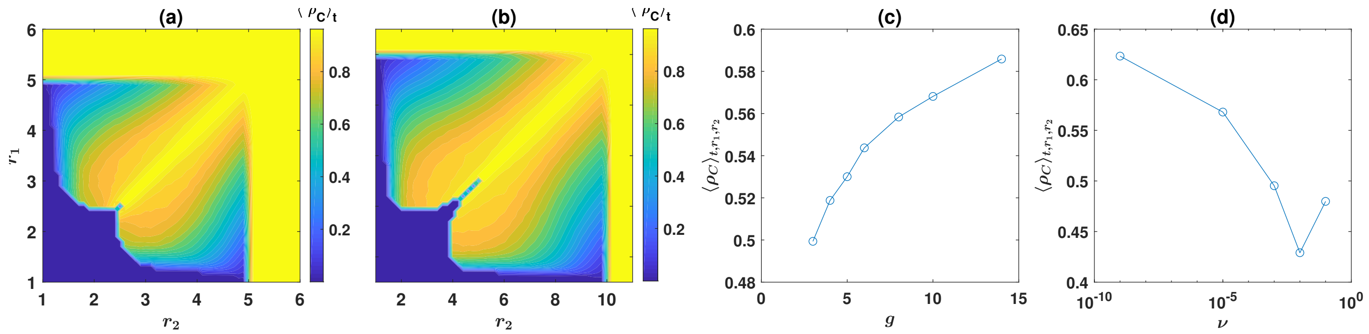

FIG. C. Dependence on group size and mutation rate. (a) and (b): (a) shows the time average density of cooperators $\langle \rho_C \rangle_t = \langle \rho_C^1 + \rho_C^2 \rangle_t$ where $g = 5$ and $\nu = 10^{-5}$ and (b) is the case of $g = 10$ and $\nu = 10^{-5}$. (c): The time average density of cooperators averaged over the phase diagram ($1 \leq r_1, r_2 \leq g$) as a function of g. As can be seen the level of cooperation increases with g. Here, $\nu = 10^{-5}$. (d) time average density of cooperators averaged over the phase diagram ($1 \leq r_1, r_2 \leq g$) as a function of ν. The level of cooperation is the lowest for a comparatively large mutation rate ($\nu = 10^{-2}$), and increases for both smaller and larger mutation rates. Here, $g = 10$ and $\pi_0 = 1$. In all the cases, the replicator equations are solved for $T = 4000$ time steps, and time averages are taken over the last $t = 2000$ time steps.

individuals prefer the same game. Here, we have chosen for the initial densities of different strategies: $\rho_C^1 = \rho_C^2 = \rho_D^2 = 0$ and $\rho_D^1 = 1$. Finally, in Fig. (B.f), the initial condition is a defection favoring one in which we have for the initial densities of the different strategies, $\rho_C^1 = \rho_C^2 = 0$ and $\rho_D^2 = \rho_D^1 = 0.5$. Comparison with the corresponding figures for the solutions of the replicator dynamics in Fig. (A), reveals a high level of agreement between the two. However, as expected, some small shifts in the positions of the phase transitions due to finite size effects are observable, especially in smaller population sizes. We return to this in the next section.

C. DEPENDENCE OF THE COOPERATION LEVEL ON THE MODEL PARAMETERS

An important question is how the cooperation level depends on the parameters of the model? We begin answering this question by addressing the dependence of the results on the population size. In Fig. (B.g), we plot the time average cooperation level in the stationary state, resulted from a simulation in a population of size $N = 500$. Here, the initial condition is a random assignment of the strategies and the preferred games. Here, $g = 10$, $\pi_0 = c = 1$, and $\nu = 10^{-3}$. As can be seen, cooperation evolves in smaller population sizes as well. Comparison with Fig. (B.c), for the case of a population of size $N = 20000$, reveals that finite size effects favor defection, such that the phase transition line for the defective fixed point to the periodic orbit shifts towards larger enhancement factors by decreasing the population size. This aspect of the model seems different from many mechanism for the evolution of cooperation, as in the case of many other models on the subject, cooperation level decreases by increasing the population size. This problem is known as the scalable cooperation problem and can be at work to undermine cooperation in large population sizes in many cases (see refs. [40,41] in the main text). In contrast, our analysis suggests having freedom to choose between different public resources, although effective in small population sizes, by solving scalable cooperation problem, can be a strong mechanism to promote cooperation in large populations.

To see how the average cooperation level depends on the population size, in Fig. (B.h), we plot the average cooperation level over the whole phase diagram (the region defined by $1 \leq r_1, r_2 \leq g$), as a function of the population size. We note that the initial conditions used in deriving this figure is a homogeneous initial condition in which the strategies and the preferred games of the individuals are randomly assigned. As can be seen, the average cooperation level increases with increasing the population size. As just described, this increment results from a shift in the position of the transition between the defective fixed point and partially cooperative periodic orbit, to smaller enhancement factors by increasing the population size.

We continue our study of the dependence of the results on the model parameters, by considering the dependence on the group size g in Fig. (C.a). Here, the contour plot of the cooperation level in the $r_1 - r_2$ plane, resulted from the numerical solution of the replicator dynamics is presented. Here, we have changed the group size to $g = 5$. As before, we have $\nu = 10^{-3}$, $c = \pi_0 = 1$, and the initial condition is a homogeneous initial condition in which the strategies and the preferred games of the individuals are randomly assigned. As expected, the same behavior, observed before for a different group size is observed here: In small enhancement factors, the dynamics settle into a defective fixed point, and a transition to the periodic orbit in which cooperators survive, occurs as the enhancement factors increase. To see how the cooperation level changes with the group size, we calculate the average cooperation level in the $r_1 - r_2$

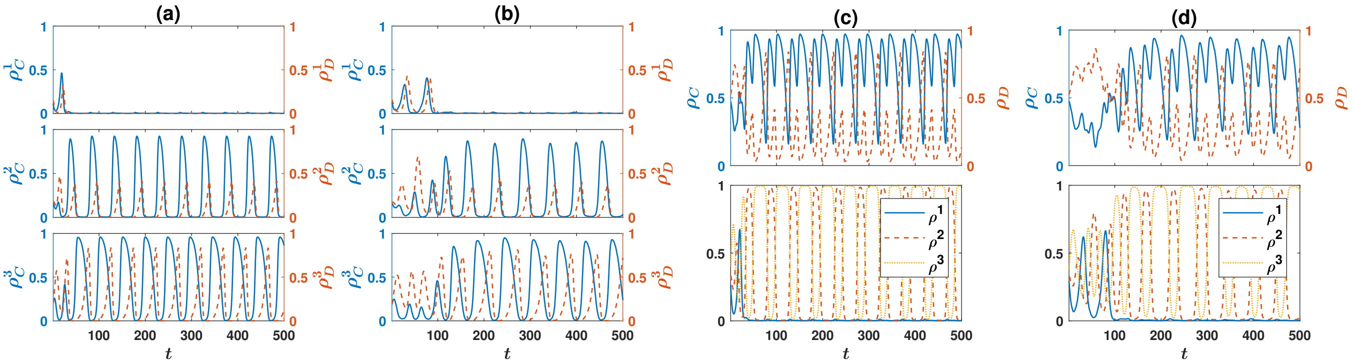

FIG. D. The dynamics of the model for $n = 3$ games. (a) and (b): The density of cooperators ρ_C^i (slid line), and defectors ρ_D^i (dashed line) who prefer game i, for $i = 1$ to $i = 3$ (from top to bottom). (c) and (d): The total density of cooperators $\rho_C = \rho_C^1 + \rho_C^2 + \rho_C^3$ (solid line) and defectors $\rho_D = \rho_D^1 + \rho_D^2 + \rho_D^3$ (dashed line) (top), and the density of individuals who prefer different games $\rho^i = \rho_C^i + \rho_D^i$, for $i = 1$ to $i = 3$ (bottom). (a) and (c) result from the replicator dynamics, and (b) and (d) result from a simulation in a population of size $N = 20000$. Here, $g = 10$, $\nu = 0.001$, $c = \pi_0 = 1$, $r_1 = 2$, $r_2 = 2.75$, and $r_3 = 3.5$. The initial condition is a homogeneous initial condition in which the strategies and the preferred games of the individuals are randomly assigned.

plane, in the interval defined by $1 \leq r_1 r_2 \leq g$, and plot this, as a function of group size g in Fig. (C.c). As can be seen the cooperation level increases with increasing the group size.

To study the dependence of the cooperation level on the mutation rate ν, in Fig. (C.b), we change the mutation rate to $\nu = 10^{-5}$, and plot the contour plot of the time average density of the cooperators $\langle \rho_C \rangle_t$, as it results from the numerical solutions of the replicator dynamics. Here, we have set $g = 10$, and $c = \pi_0 = 1$. The initial condition is a homogeneous initial condition in which the density of all the strategies are the same. By comparison with the case of $\nu = 10^{-3}$ studied before, we note that by decreasing the mutation rate, the phase transition line from the defective fixed point to the periodic orbit, shifts to smaller enhancement factors (this can be more obviously seen in Figure (1.c) in the main text, where the phase diagram of the model for two different mutation rates are plotted). The shifts in the phase transition lines, result in the enhancement of the cooperation level for smaller mutation rates. This can be seen in Fig. (C.d), where the average density of the cooperators in the $r_1 - r_2$ plane, in the region $1 \leq r_1, r_2 \leq g$, as a function of the mutation rate is plotted. Here, as can be seen, the cooperation level decreases with increasing the mutation rate. However, for very large mutation rates, the cooperation level increases. This is due to the fact that for very large mutation rates, the density of the cooperators maintained in the system due to random mutations is higher than the density of the cooperators maintained in the population as a result of the competition between public resources.

D. AN EXAMPLE OF THE DYNAMICS OF THE MODEL IN THE CASE OF N = 3 GAMES

As it is made explicit in the main text, the model can be defined in the general context where $n \geq 2$ PGGs exist and compete to attract individuals. Here, to shed light on the dynamics of the model in the case that $n > 2$ public resources exist, we consider a case with $n = 3$ public resources, and study the dynamics of the model, using the replicator dynamics and simulations.

Similarly to the $n = 2$ case, the dynamics with $n = 3$ can settle in a fixed point or a periodic orbit. We plot the density of cooperators ρ_C^i, and defectors ρ_D^i, who prefer game i, for $i = 1$ to $i = 3$ (from top to bottom), in Fig. (D.a) and Fig. (D.b). Fig. (D.a) shows the result of the replicator dynamics, and Fig. (D.b) results from a simulation in a population of size $N = 10000$. The total density of cooperators $\rho_C = \rho_C^1 + \rho_C^2 + \rho_C^3$, and the total density of defectors $\rho_D = \rho_D^1 + \rho_D^2 + \rho_D^3$ are plotted in the top panels of Fig. (D.c) (replicator dynamics) and Fig. (D.d) (simulations). The density of the individuals who prefer game i is plotted in the bottom panels of Fig. (D.c) (replicator dynamics) and Fig. (D.d) (simulations). Here, $g = 10$, $\nu = 0.001$, $r_1 = 2$, $r_2 = 2.75$, and $r_3 = 3.5$. The initial condition is a homogeneous initial condition in which the strategies and the preferred games of the individuals are randomly assigned.

As can be seen in the figure, the densities of individuals in different public resources shows fluctuations and on average is higher in the higher quality resource. This results from the fact that during a cycle a high quality resource attracts and maintains individuals for a longer period of time compared to the low quality resources. However, when a high quality resource becomes occupied with a high density of cooperators, defectors start to grow in that resource.

This decreases the profitability of the high quality resource such that eventually cooperators take shelter in a lower quality resource and make it the more profitable resource. We note that, for the values of the enhancement factors used here, the lowest quality resource (game 1, with $r_1 = 2$) becomes almost obsolete in the stationary state. However, this is not necessarily the case, and it can happen that all the resources attract a significant number of individuals, provided their enhancement factors are very close.

E. COMPUTER CODES

In this section, the MATLAB codes used in this study are presented. In Sec. (E.A), MATLAB functions used for the numerical solution of the replicator dynamics and simulation in the case of $n = 2$ games are presented. And in Sec. (E.B), MATLAB functions used for the numerical solution of the replicator dynamics and simulation in the case of $n = 3$ games are presented.

E.A. The case of $n = 2$ games

Numerical solutions of the replicator dynamics

```
%This function numerically solves the replicator dynamics of the model for the case of n = 2 games.
Outputs: rhoc1t and rhoc2t are, respectively, the density of cooperators in game 1 and game 2. rhod1tand rhod2t are, respectivly, the density of defectors in game 1, game 2.
Inputs: T is the simulation duration, Ti is the initial time to record the data, g is the group size, r1 and r2 are the enhancement factors of, respectively, resource 1 and resource 2. base is the base payoff, and cost is the cost of investment in public resource nus mutation rate in strategies, and nug is the emutatation rate in game preferrence. rhoc1, rhod1, rhoc2, and rhod2 are the intial conditions (should add up to 1), and caltime is the time intervall for recording the data.
function [ rhoc1t , rhoc2t , rhod1t , rhod2t ] = fmftwogamesInitial ( T , Ti , g , r1 , r2 , cost , base , nus , nug , rhoc1 , rhod1 , rhoc2 , rhod2 , caltime )
  tr=0;
  for t=1:T;
      %defining the payoffs of different strategies
      pic1=0;pid1=0;pic2=0;pid2=0;
      %density of individuals in game 1
      rho1=rhoc1+rhod1;
      %density of individuals in game 2
      rho2=rhoc2+rhod2;
      %calculating the average payoff of those who prefer game 1
      for Npc=0:g-1;
         for Npd=0:g-Npc-1;
            Nnp=g-1-Npc-Npd;
            prob=fp2(Npc,Npd,Nnp,rhoc1,rhod1,1-rho1,g);
            Ntot=Npc+Npd;
            pic1=pic1+(r1*(Npc+1)/(Ntot+1))*prob;
            pid1=pid1+(r1*(Npc)/(Ntot+1))*prob;
         end
      end
      %calculating the average payoff of those who prefer game 2
      for Npc=0:g-1;
         for Npd=0:g-Npc-1;
            Nnp=g-1-Npc-Npd;
            prob=fp2(Npc,Npd,Nnp,rhoc2,rhod2,1-rho2,g);
            Ntot=Npc+Npd;
            pic2=pic2+(r2*(Npc+1)/(Ntot+1))*prob;
            pid2=pid2+(r2*(Npc)/(Ntot+1))*prob;
         end
      end
      %final payoffs
```

```
            pic1=base+pic1+(-cost);
            pid1=base+pid1;
            pic2=base+pic2+(-cost);
            pid2=base+pid2;
            %average population payoff
            mp=rhoc1*pic1+rhod1*pid1+rhoc2*pic2+rhod2*pid2;
            %The density of strategies before mutation
            drhoc1=rhoc1*(pic1/mp);
            drhod1=rhod1*(pid1/mp);
            drhoc2=rhoc2*(pic2/mp);
            drhod2=rhod2*(pid2/mp);
            %The density of strategies after mutation
           rhoc1=drhoc1*(1-nus-nug+nus*nug)+(drhoc2)*(nug-nus*nug)+(drhod1)*(nus-nus*nug)+(drhod2)*(nus*nug);
           rhod1=drhod1*(1-nus-nug+nus*nug)+(drhod2)*(nug-nus*nug)+(drhoc1)*(nus-nus*nug)+(drhoc2)*(nus*nug);
           rhoc2=drhoc2*(1-nus-nug+nus*nug)+(drhoc1)*(nug-nus*nug)+(drhod2)*(nus-nus*nug)+(drhod1)*(nus*nug);
           rhod2=drhod2*(1-nus-nug+nus*nug)+(drhod1)*(nug-nus*nug)+(drhoc2)*(nus-nus*nug)+(drhoc1)*(nus*nug);
            %Recording the data
            if mod(t,caltime)==0 && t>=Ti
            tr=tr+1;
                rhoc1t(tr)=rhoc1;
                rhod1t(tr)=rhod1;
                rhoc2t(tr)=rhoc2;
                rhod2t(tr)=rhod2;
            end
        end
    end
```

```
%% This function calculates the probability that a group composed of, n1, n2, and n3 individuals, who are found in the population with densities, respectively, rho1, rho2, and rho3.
function [prob]=fp2(n1,n2,n3,rho1,rho2,rho3,g)
   prob = ( factorial(g-1) / ( factorial(n1) * factorial(n2) * factorial(n3) ) ) *
   (rho1^n1) * (rho2^n2) * (rhovcd^nvcd);
end
```

Simulation

% This function simulates the dynamics of the model for the case of $n = 3$ games.
Outputs: mc1 and mc2 are respectively, the density of cooperators in game 1 and game 2. md1 and md2 are respectivly, the density of defectors in game 1 and game 2.
Inputs: T is the simulation duration, N the population size, groupsize is the group size, nus mutation rate in strategies, and nug is the emutatation rate in game preferrence. mulrate1 and mulrate2 are the enhancement factors of, respectively, resource 1 and resource 2. base is the base payoff. cost is the cost of investment in public resource, and caltime is the time intervall for recording the data.

```
   function [mc1 , mc2 , md1 , md2] = ftwogamesInRand(T , N , groupsize , nus , nug , mulrate1 , mulrate2, base ,
   cost , caltime)
   % Setting the initial conditions for strategy
   strategy=randi(2,N,1)-1;
   % Setting the initial conditions for the preferred game
   playstrategy=randi(2,N,1)-1;
   tr=0;
   for t=1:T;
        payoff=base*ones(N,1);
        popvec=randperm(N);
        while length(popvec)>=groupsize;
            % Randomely drawing groups of groupsize individuals.
            group=popvec(1:groupsize);
            popvec(1:groupsize)=[];
```

```
    % Those who prefer game 1.
    g1=group(playstrategy(group)==0);
    % Those who prefer game 2.
    g2=group(playstrategy(group)==1);
    wealth1=mulrate1*sum(strategy(g1))*cost;
    wealth2=mulrate2*sum(strategy(g2))*cost;
    len1=length(g1);
    len2=length(g2);
    if len1>0;
        % Payoff attribution in game 1.
        payoff(g1)=payoff(g1)+wealth1/len1-cost*strategy(g1);
    end
    if len2>0;
        % Payoff attribution in game 2.
        payoff(g2)=payoff(g2)+wealth2/len2-cost*strategy(g2);
    end
end;
payofft=payoff;
strategy2=strategy;
strategyplay2=playstrategy;
% Setting negative payoffs equal to zero. This is executed only when
% the base payoff is smaller that the cost of cooperation.
payofft(payofft<0)=0;
if sum(payofft)==0;
    payofft=ones(1,N);
end;
payofft=(payofft)/sum(payofft);
% The whole population is updated. For each individual in the next generation and individual is selected as
parent with a probability proportional to its payoff.
for it=1:N;
    tesr=0;
    tesr2=rand(1);
    % As the parent of individual it, the parent, winner, is selected with a probability proportional to its payoff
    for ie=1:N;
        tesr=tesr+payofft(ie);
        if tesr >= tesr2;
            winner=ie;
            break;
        end;
    end;
    % Offspring inherits the strategy and the preferred game of its parent
    strategy2(it)=strategy(winner);
    strategyplay2(it)=playstrategy(winner);
    % Mutation in strategy
    if nus>rand(1);
        strategy2(it)=1-strategy2(it);
    end;
    % Mutation in the preferred game
    if nug>rand(1);
        strategyplay2(it)=1-strategyplay2(it);
    end;
end
strategy=strategy2;
playstrategy=strategyplay2;
% recording the data
if mod(t,caltime)==0
    tr=tr+1;
    mc1(tr)=sum(strategy(playstrategy==0)==1);
```

```
        md1(tr)=sum(strategy(playstrategy==0)==0);
        mc2(tr)=sum(strategy(playstrategy==1)==1);
        md2(tr)=sum(strategy(playstrategy==1)==0);
    end
end
mc1=mc1/N;md1=md1/N;mc2=mc2/N;md2=md2/N;
```

E.B. The case of $n = 3$ games

Numerical solutions of the replicator dynamics

```
% This function numerically solves the replicator dynamics of the model for the case of n = 3 games.
Outputs: rhoc1t, rhoc2t, and rhoc3t are respectively, the density of cooperators in game 1, game 2, and game 3. rhod1t, rhod2t, and rhod3t are respectivly, the density of defectors in game 1, game 2 and game 3.
Inputs: T is the simulation duration, Ti is the initial time to record the data, g is the group size, r1, r2, and r3 are the enhancement factors of, respectively, resource 1, resource 2, and resource 3. base is the payoff, and cost is the cost of investment in public resource nus mutation rate in strategies, and nug is the emutatation rate in game preferrence. rhoc1, rhod1, rhoc2, rhod2, rhoc3, rhod3, are the intial conditions (should add up to 1), and caltime is the time intervall for recording the data.
function [ rhoc1t , rhoc2t , rhoc3t , rhod1t , rhod2t , rhod3t ] = fmfthreegamesInitial ( T , Ti , g , r1 , r2 , r3 , cost , base , nus , nug , rhoc1 , rhod1 , rhoc2 , rhod2 , rhoc3 , rhod3 , caltime )
 tr=0;
 for t=1:T;
    %defining the payoffs of different strategies
    pic1=0;pid1=0;pic2=0;pid2=0;pic3=0;pid3=0;
    %density of individuals in game 1
    rho1=rhoc1+rhod1;
    %density of individuals in game 2
    rho2=rhoc2+rhod2;
    %density of individuals in game 3
    rho3=rhoc3+rhod3;
    %calculating the average payoff of those who prefer game 1
    for Npc=0:g-1;
        for Npd=0:g-Npc-1;
            Nnp=g-1-Npc-Npd;
            prob=fp2(Npc,Npd,Nnp,rhoc1,rhod1,1-rho1,g);
            Ntot=Npc+Npd;
            pic1=pic1+(r1*(Npc+1)/(Ntot+1))*prob;
            pid1=pid1+(r1*(Npc)/(Ntot+1))*prob;
        end
    end
    %calculating the average payoff of those who prefer game 2
    for Npc=0:g-1;
        for Npd=0:g-Npc-1;
            Nnp=g-1-Npc-Npd;
            prob=fp2(Npc,Npd,Nnp,rhoc2,rhod2,1-rho2,g);
            Ntot=Npc+Npd;
            pic2=pic2+(r2*(Npc+1)/(Ntot+1))*prob;
            pid2=pid2+(r2*(Npc)/(Ntot+1))*prob;
        end
    end
    %calculating the average payoff of those who prefer game 3
    for Npc=0:g-1;
        for Npd=0:g-Npc-1;
            Nnp=g-1-Npc-Npd;
            prob=fp2(Npc,Npd,Nnp,rhoc3,rhod3,1-rho3,g);
            Ntot=Npc+Npd;
```

```
            pic3=pic3+(r3*(Npc+1)/(Ntot+1))*prob;
            pid3=pid3+(r3*(Npc)/(Ntot+1))*prob;
        end
    end
    %final payoffs
    pic1=base+pic1+(-cost);
    pid1=base+pid1;
    pic2=base+pic2+(-cost);
    pid2=base+pid2;
    pic3=base+pic3+(-cost);
    pid3=base+pid3;
    %average population payoff
    mp=rhoc1*pic1+rhod1*pid1+rhoc2*pic2+rhod2*pid2+rhoc3*pic3+rhod3*pid3;
    %The density of strategies before mutation
    drhoc1=rhoc1*(pic1/mp);
    drhod1=rhod1*(pid1/mp);
    drhoc2=rhoc2*(pic2/mp);
    drhod2=rhod2*(pid2/mp);
    drhoc3=rhoc3*(pic3/mp);
    drhod3=rhod3*(pid3/mp);
    %The density of strategies after mutation
    rhoc1=drhoc1*(1-nus-nug+nus*nug)+(drhoc2)*(nug-nus*nug)/2+(drhoc3)*(nug-nus*nug)/2+(drhod1)*(nus-
    nus*nug)+(drhod2)*(nus*nug)/2+(drhod3)*(nus*nug)/2;
    rhod1=drhod1*(1-nus-nug+nus*nug)+(drhod2)*(nug-nus*nug)/2+(drhod3)*(nug-nus*nug)/2+(drhoc1)*(nus-
    nus*nug)+(drhoc2)*(nus*nug)/2+(drhoc3)*(nus*nug)/2;
    rhoc2=drhoc2*(1-nus-nug+nus*nug)+(drhoc1)*(nug-nus*nug)/2+(drhoc3)*(nug-nus*nug)/2+(drhod2)*(nus-
    nus*nug)+(drhod1)*(nus*nug)/2+(drhod3)*(nus*nug)/2;
    rhod2=drhod2*(1-nus-nug+nus*nug)+(drhod1)*(nug-nus*nug)/2+(drhod3)*(nug-nus*nug)/2+(drhoc2)*(nus-
    nus*nug)+(drhoc1)*(nus*nug)/2+(drhoc3)*(nus*nug)/2;
    rhoc3=drhoc3*(1-nus-nug+nus*nug)+(drhoc1)*(nug-nus*nug)/2+(drhoc2)*(nug-nus*nug)/2+(drhod3)*(nus-
    nus*nug)+(drhod1)*(nus*nug)/2+(drhod2)*(nus*nug)/2;
    rhod3=drhod3*(1-nus-nug+nus*nug)+(drhod1)*(nug-nus*nug)/2+(drhod2)*(nug-nus*nug)/2+(drhoc3)*(nus-
    nus*nug)+(drhoc1)*(nus*nug)/2+(drhoc2)*(nus*nug)/2;
    %Recording the data
    if mod(t,caltime)==0 && t>=Ti
    tr=tr+1;
        rhoc1t(tr)=rhoc1;
        rhod1t(tr)=rhod1;
        rhoc2t(tr)=rhoc2;
        rhod2t(tr)=rhod2;
        rhoc3t(tr)=rhoc3;
        rhod3t(tr)=rhod3;
    end
  end
end

%% This function calculates the probability that a group composed of, n1, n2, and n3 individuals, who are found in
the population with densities, respectively, rho1, rho2, and rho3.
function [prob]=fp2(n1,n2,n3,rho1,rho2,rho3,g)
  prob = ( factorial(g-1) / ( factorial(n1) * factorial(n2) * factorial(n3) ) ) *
  (rho1^n1) * (rho2^n2) * (rhovcd^nvcd);
end
```

Simulation

```
% This function simulates the dynamics of the model for the case of n = 3 games.
```

Outputs: mc1, mc2, and mc3 are respectively, the density of cooperators in game 1, game 2, and game 3. md1, md2, and md3 are respectivly, the density of defectors in game 1, game 2 and game 3.
Inputs: T is the simulation duration, N the population size, groupsize is the group size, nus mutation rate in strategies, and nug is the emutatation rate in game preferrence. mulrate1, mulrate2, and mulrate3 are the enhancement factors of, respectively, resource 1, resource 2, and resource 3. base is the base payoff. cost is the cost of investment in public resource, and caltime is the time interval for recording the data.

```
function [mc1 , mc2 , mc3 , md1 , md2 , md3] = fthreegamesInRand(T , N , groupsize , nus , nug , mulrate1 ,
mulrate2 , mulrate3 , base , cost , caltime)
% Setting the initial conditions for strategy
strategy=randi(2,N,1)-1;
% Setting the initial conditions for the preferred game
playstrategy=randi(3,N,1)-1;
tr=0;
for t=1:T;
    payoff=base*ones(N,1);
    popvec=randperm(N);
    while length(popvec)>=groupsize;
        % Randomely drawing groups of groupsize individuals.
        group=popvec(1:groupsize);
        popvec(1:groupsize)=[];
        % Those who prefer game 1.
        g1=group(playstrategy(group)==0);
        % Those who prefer game 2.
        g2=group(playstrategy(group)==1);
        % Those who prefer game 3.
        g3=group(playstrategy(group)==2);
        wealth1=mulrate1*sum(strategy(g1))*cost;
        wealth2=mulrate2*sum(strategy(g2))*cost;
        wealth3=mulrate3*sum(strategy(g3))*cost;
        len1=length(g1);
        len2=length(g2);
        len3=length(g3);
        if len1>0;
            % Payoff attribution in game 1.
            payoff(g1)=payoff(g1)+wealth1/len1-cost*strategy(g1);
        end
        if len2>0;
            % Payoff attribution in game 2.
            payoff(g2)=payoff(g2)+wealth2/len2-cost*strategy(g2);
        end
        if len3>0;
            % Payoff attribution in game 3.
            payoff(g3)=payoff(g3)+wealth3/len3-cost*strategy(g3);
        end
    end;
    payofft=payoff;
    strategy2=strategy;
    strategyplay2=playstrategy;
    % Setting negative payoffs equal to zero. This is executed only when
    % the base payoff is smaller that the cost of cooperation.
    payofft(payofft<0)=0;
    if sum(payofft)==0;
        payofft=ones(1,N);
    end;
    payofft=(payofft)/sum(payofft);
    % The whole population is updated. For each individual in the next generation and individual is selected as
    parent with a probability proportional to its payoff.
    for it=1:N;
```

```
        tesr=0;
        tesr2=rand(1);
        % As the parent of individual it, the parent, winner, is selected with a probability proportional to its payoff
        for ie=1:N;
            tesr=tesr+payofft(ie);
            if tesr >= tesr2;
                winner=ie;
                break;
            end;
        end;
        % Offspring inherits the strategy and the preferred game of its parent
        strategy2(it)=strategy(winner);
        strategyplay2(it)=playstrategy(winner);
        % Mutation in strategy
        if nus>rand(1);
            strategy2(it)=1-strategy2(it);
        end;
        % Mutation in the preferred game
        if nug>rand(1);
            aa=randi(3)-1;
            while aa==strategyplay2(it);
                aa=randi(3)-1;
            end
            strategyplay2(it)=aa;
        end;
    end
    strategy=strategy2;
    playstrategy=strategyplay2;
    % recording the data
    if mod(t,caltime)==0
        tr=tr+1;
        mc1(tr)=sum(strategy(playstrategy==0)==1);
        md1(tr)=sum(strategy(playstrategy==0)==0);
        mc2(tr)=sum(strategy(playstrategy==1)==1);
        md2(tr)=sum(strategy(playstrategy==1)==0);
        mc3(tr)=sum(strategy(playstrategy==2)==1);
        md3(tr)=sum(strategy(playstrategy==2)==0);
    end
end
mc1=mc1/N;md1=md1/N;mc2=mc2/N;md2=md2/N;mc3=mc3/N;md3=md3/N;
```
