# Supplementary material for: Freedom to choose between public resources promotes cooperation
Source: PLoS Comput Biol. 2021 Feb 8;17(2):e1008703. doi: 10.1371/journal.pcbi.1008703 (PMC7895419; doi:10.1371/journal.pcbi.1008703)
Supplement: Attachment — Submitted filename: response.pdf [file pcbi.1008703.s002.pdf]

Responses to review comments

I am thankful to all the reviewers and the editors for careful reading of the manuscript. In the following, I provide my point by point responses to the review comments.

Response to Guest editor's suggestions

Guest editor:
Thank you for your manuscript. In the meanwhile, it has been seen by three experts in evolutionary game theory and dynamical systems. They all agree that the manuscript is sound and that it describes an interesting mechanism for cooperation. Based on my own reading, I fully agree. The reviewers also make a number of very constructive suggestions for how to further improve the paper. Please take all these suggestions into account when preparing a revised version.
Let me briefly mention two further suggestions.
Response:
I am thankful to the guest editor for his careful reading and assessment of the manuscript and for his constructive suggestions.
Guest editor:
First, it seems to me the present setup is better explained by saying that group members have a choice between different public good games to play, instead of saying that different public resources "compete". I find the term "competition" somewhat misleading. It suggests the resources themselves have strategic motives.
Response:
I agree, there is no direct competition between different public goods. What is essential is having a choice between public goods, and the competition between public goods is only implicit. Based on this suggestion, I have changed the name of the manuscript, and revised the text accordingly to make this point more salient.
Guest editor:
Second, upon my first reading, I had some problems to clearly see which mechanism prevents cooperation from going extinct in this model. Only after closer inspection of Eq. (3), I realized that it's crucial that the present model allows for smaller effective group sizes. That is, even if the baseline group size is g, the actual group in which a player interacts can be considerably smaller. If the effective group size is sufficiently small (i.e., smaller than the respective multiplication factor r_i), cooperation becomes a dominant strategy. All of this is implicit in the payoff Eq. (3), but I would appreciate if the author mentions this aspect more explicitly. Please also note that in Eq. (3), the term with r_i needs to be multiplied by c (note that for the simulations this is inconsequential, because there the parameter c has been set to one).
Response:
In the discussion section, I have added a discussion about the intuition behind the mechanism where this point, rightly observed by the guest editor, is explained.
I thank the guest editor for bringing my attention to a typo in eq. (3). This is now corrected in the revised manuscript.

Response to Reviewer N. 1

Reviewer N. 1:
This paper deals with the evolution of cooperation in public goods games (PGG) when players can

choose between multiple public resources to contribute to. Individuals not only have a (deterministic) strategy for the PGG, but also a preferred game they want to play. In each time step, random groups of a certain size are formed from the population. Players enter their preferred game, interact according to their strategy, and receive the corresponding payoff and an additional base payoff. Then, the population reproduces, subject to mutations. The authors show that the competition between different public goods games can help cooperation evolve. This is due to the fact that cooperators can find shelter and survive in the less profitable resource while defectors predominantly join the higher quality resource. Cooperators can then outcompete the defectors, and this effect is strongest when the public resources have similar quality, i.e. enhancement factors r. Solving the replicator dynamics of this system, the authors find two possible attractors depending on initial conditions and the different qualities of the public resources: either a fixed point or a limit cycle. In the latter case, cooperators are preserved and cyclically dominate the population. Meanwhile, for too small enhancement factors, the dynamics settles in a fixed point where defection dominates. The paper then further explores the system's dependence on the different enhancement factors in more detail, additionally supported by simulations. Robustness results are also provided in the Supplementary Information. The authors come to the conclusion that this mechanism for the evolution of cooperation does not suffer from the decrease in cooperation rate as group size gets larger, which is a contrast to the corresponding observation in classic PGGs.
The paper and its results are interesting, and its motivation clear, which is well explained already in the abstract. Also, the good match between simulations and analytical results inspires confidence. However, I find some issues with this work that should be addressed before publication.

Response:

I am thankful to the reviewer for his careful reading and assessment of the manuscript, for his constructive comments, and for finding the manuscript of interest.

Reviewer N. 1:

For one, I am a bit confused regarding the definition of the densities of cooperators and defectors, respectively. What is the quantity that these densities are taken relative to? This is never made quite clear, and I somehow would have expected that the density of cooperators and the density of defectors should add up to one, considering there is no other strategy at play? Instead, Fig. 1(b) does not look like this is true, which is puzzling to me. Also, how is the density of cooperators with respect to one game type defined? It is hard to interpret the figures without that information. Furthermore, can one conceptually even add up cooperation in the two (or more) different games to get a global "cooperation density"? I would appreciate some insight on this.

Response:

The density of cooperators (defectors) in game i, ρ_C^i (ρ_D^i), is defined as the fraction of individuals who prefer game i and cooperate (defect). The (total) density of cooperators is defined as the sum of the density of cooperators in all the games, $\rho_C = \sum_{i=1}^n \rho_C^i$. Similarly the density of defectors is defined as the sum of the density of defectors in all the games, $\rho_D = \sum_{i=1}^n \rho_D^i$. As the reviewer expects, the the total density of cooperators and defectors add up to 1, $\rho_C + \rho_D = 1$. This holds in Fig. 1(b). I note that here the blue solid line is the density of cooperators. The density of defectors is not shown, as it is easily inferrable using the relation $\rho_C = 1 - \rho_C$. The red dashed line is NOT the density of defectors. It is the density of individuals in game 1. While the y-axis label was correct in the old version of the manuscript, the caption and main text mistakenly asserted that this is the density of defectors which caused confusion. This is corrected now. In addition, I have now explicitly defined the density of cooperators and defectors in game i, the total density of cooperators and defectors, and the density of individuals in game i. I hope the text is clear now. I am thankful to the reviewer for bringing my attention to this error.

It seems adding up the density of cooperator in different games to be conceptually consistent. To

my judgment, this brings a proper measure of the extent of cooperative behavior in the system. Other variables, such as the density of cooperators in each game are not always capable of giving an overall picture of cooperation level in the system. The virtue of defining the total cooperation level is more appreciated when going to higher number of public resources, for example $n = 3$.
Of course, by looking at the system only through the lens of this quantity, some important aspects will not be visible. For this reason, wherever I used this quantity, I have also tried to use other complementary quantities, such as the density of cooperators in each game to clarify other aspects.

Reviewer N. 1:

Another question I have is the relation of these results to the results by Hauert et al. in Reference [5], which deals with volunteering/abstaining in the PGG. The authors of that work show that the option to abstain can give rise to cooperation in a rock-paper-scissors cycle, as cooperators start to abstain when defector numbers rise, which in turn enables small groups of loners to switch back to cooperation. Considering that one could see cooperators switching their preferred game is a kind of "abstaining", I would be curious to see a comparison of the two papers in a bit more detail than is currently provided.

Response:

Based on this fruitful suggestion, a comparison of the two mechanisms, where similarities and differences are mentioned, is now provided in the discussion section.

Reviewer N. 1:

Furthermore, it would be important to streamline the paper, such that it flows better, and also remove typos and grammatical mistakes at the same time. E.g., in the results section, crucial statements are repeated multiple times, which certainly reminds the reader of the main message of the paper, but inhibits the reading flow quite a bit. It would be good to give this section a bit more structure. As for typos, e.g. in the author summary, the first sentence is out of order, and in Fig.1, the right y-axis label of panel (b) is off. Also, there is a stray "Lorem ipsum" on page 8 in the paragraph "S2 Fig."

Response:

I have spent some time to give the result section more structure. It is now divided into three subsections. It is tried to avoid repetition. At least one paragraph (in subsection cooperation level) is majorly revised and some parts alongside panel 1.d in the old version which was referenced in this part, are excluded to avoid repetition. Based on Reviewer N. 3's suggestion, the case of $n = 3$ games is brought from the supplementary material (after edits) into the result section.
In the author summary, I chose to start the paragraph by posing a question, an answer to which is then introduced in the paragraph. As mentioned before, the right y-axis label of panel 1.b was correct, but the caption was wrong, which caused confusion. This is corrected now. I thank the reviewer for bringing my attention the "Lorem ipsum". It is removed now. Time is spent to remove typos and improve the language.

Reviewer N. 1:

Minor comments:
(-) One pretty important mechanism that gives rise to cooperation in social dilemmas is direct reciprocity (repeated interactions). Why is this not mentioned?

Response:

I thank the reviewer for reminding me of this point. In the revised manuscript direct reciprocity is mentioned as well. The reason behind its omission in the old version was that most of the work on direct reciprocity consider binary interactions, that is the Prisoner's Dilemma. In extending to more than two players, that is in public goods game, direct reciprocity can meet some difficulties in promoting cooperation. The problem originates from the fact that in a public goods game, retaliation against a defector, punishes all the group members, cooperators and defectors alike. For this reason, arguably, it is not as effective in group interactions as it is in binary ones. However, it is still fair to

say direct reciprocity is an important mechanism and deserves mentioning even in the case of group interactions.

Response to Reviewer N. 2

Reviewer N. 2:
In "Competition between public resources promotes cooperation" authors show that competition between public resources provides a simple mechanism for cooperation. In particular, since defectors might be tempted to join the highest quality resource pool, cooperators get a window of opportunity by using lower quality resources. Not surprisingly, this mechanism works best when the qualities of the two highest quality resources are similar, which is also when cooperation is most abundant.

The proposed model is intuitive and clear, and I agree it can be used in many biological and social contexts where individuals can form different groups or join different institutions to perform a collective task. In general, how and why cooperation emerges in the public goods game is an intensely investigated subject with obvious practical ramifications. Methods of computational biology, network science, and statistical physics have been applied successfully and with much effect recently to shed light on the problem from many different perspectives, and also to outline many different ways on how cooperation could be promoted. In this sense, the study certainly addresses a relevant setup, and it also delivers results that might be of interest to the readership of PLOS Computational Biology.

I am in general in favor of publication subject to taking into account the following comments with care and love to detail.

Response:
I am thankful to the reviewer for his careful reading and assessment of the manuscript, and for his constructive comments.

Reviewer N. 2:
I would advise against explicitly claiming novelty in the abstract and elsewhere. Similar mechanisms have been observed before, so this is hardly the first time we see that competition for limited resources, if presented rightly, can foster cooperation.

One related paper from 2014 is Excessive abundance of common resources deters social responsibility, Sci. Rep. 4, 4161 (2014), which I am sure would fit very well to the introduction, especially related to the importance of the competition the author is focusing on. A more recent paper also focusing on resources in public cooperation is Rewarding endowments lead to a win-win in the evolution of public cooperation and the accumulation of common resources, Chaos, Solitons & Fractals 134, 109694 (2020). A popular review for the public goods game is also Evolutionary dynamics of group interactions on structured populations: A review, J. R. Soc. Interface 10, 20120997 (2013).

Response:
I have revised the text, to discuss connection with other works. This includes competition between resources which is now mentioned in the introduction, and in the empirical context, also in discussion. Another part of the result concerns the effect that freedom to choose between public resources have on promotion of cooperation. The connection of this aspect with abstaining is also discussed in the discussion. The abstract is also revised.

I thank the reviewer for bringing my attention to these works, which I have tried to benefit from in the revision.

Reviewer N. 2:
I wonder about the robustness of the presented results in structured populations. Can the author please comment on that.

Response:
I have looked at this for the case of n=2 games, but not in full depth. As having a choice between

public resources promotes cooperation in a well-mixed population, and given that the evolution of cooperation in public goods game is easier in structured populations, it might be intuitively expected this mechanism to be able to promote cooperation in structured populations as well. Simulations suggest this is the case. More-ever, the evolution of cooperation seems to be facilitated in this case, in the sense that cooperation can evolve for smaller enhancement factors, compared to a situation where only one public resource exists. In the revised manuscript, I have commented on this in the discussion section, as a possible direction for future research.

Reviewer N. 2:

The last section should become a discussion, where not only the results are reiterated, but where also some time in spent on where we could actually expect they might be useful. Here the author can spell out many biological and social contexts where individuals can form different groups or join different institutions to perform a collective task that he mentions in the abstract and introduction.

Response:

I thank the reviewer for this constructive suggestion. This is done in the revised manuscript. Benefiting from constructive comments from the reviewers and the editor, in the discussion, some examples where the model can be useful, such as in microorganism, group living animals, and human societies, and connection with other works regarding both beneficial effect of competition and abstaining is discussed. In addition, the intuition behind the mechanism and a possible direction for future research is discussed.

Reviewer N. 2:

I also notice some "Lorem ipsum" in the figure captions, which should be double checked and corrected.

References need a careful check for correct punctuation, capitalization, and overall accuracy.

Response:

I thank the reviewer for bringing my attention to this typo. I detected one Lorem ipsum in the description of S2 Fig. which is removed now. Some references are added and all of the references are checked for accuracy.

Response to Reviewer N. 3

Reviewer N. 3:

This work provides an insight into the emergence of cooperation in the public's good game (in an evolutionary context). More specifically, the author explores the effect that an enhancement factor has on the emergence of cooperation, in a population of cooperators and defectors.

The author considers a population where individuals are divided into groups. Each individual can choose a strategy and a game. Each game is a publics good game of n players. The difference between the games is the enhancement factor. All the contributions at the end of the round are multiplied by an enhancement factor and are distributed equally among the players.

The author presented results in the case of n=2 and in the case of two different enhancement factors, where commonly one is larger. The results are based on analytical calculations (using the replicator-mutation dynamics equation) and simulations. These show that:

- Defectors tend to play the game with the highest enhancement factor which allows cooperators to survive.

- Cooperation level is maximized when the enhancement factors have the same value.

Furthermore, the author includes some discussion on results for the case of n=3 and an analysis on the robustness of the results for other parameter values.

This work does provide interesting insights to the public's good game. Its a simple yet interesting formulation that allows the author to show that their mechanism can allow cooperation to occur.

However, it does have some issues, and for this reason I cannot recommend publication in its present form. More specifically:

Response:

I am thankful to the reviewer for his careful reading and assessment of the manuscript, for his constructive comments, and for finding the manuscript of interest.

Reviewer N. 3:

Material and Methods section.

- The author needs a reference for the replicator-mutation dynamics equation. - The author needs to add at least a sentence explaining Eq. (1). - Before explaining Eq. (3) the author states that j is used for the public resources, then produces the explanation using i.

Response:

A reference for the replicator-mutation dynamics is added, and it is briefly explained. The notation is changed to make it more uniform.

Reviewer N. 3:

The quality of the graphs.

In Figure 1, the author uses dotted lines and solid lines to distinguish between the density of defectors and cooperators. The size of the sub-figures make it hard to see the different line styles. I propose that Figure 1 should include only subfigures 1a and 1b. Moreover, the author uses color for the axis. If printed in black and white it is not visible. I propose that the author uses a legend instead. Also, Figure 1b, the label of the right y axis is incorrect.

The author uses the line color of Figures 1c and 1d to explain the results in the text. This should be avoided. The reader might have the manuscript in black or white, or simply is colorblind. The figures should be bigger, just have these two sub figures as Figure 2, and use different line styles.

Response:

Based on the reviewer's suggestion, panels 1a and 1b are turned into a Fig. 1, and the phase diagrams are turned into another figure, Fig. 2. The size of the markers in the phase diagram are increased to make different lines discernible independently of their colors. The label of the y axis in Fig 1b in the old version was not incorrect; The caption was. Now the text and caption are corrected. I have chosen to keep right and left axis, as I feel this looks better than having a legend. However, I have edited the line styles and referred to the line styles in the caption and text, wherever this figure is referenced, so the reader can process all the information without needing the colors.

Reviewer N. 3:

Data and computer code.

The author states that All data will be made available online after acceptance. I encourage the author to make the data available and reference them in the paper. Furthermore, I would like to encourage the author to make the computer code for the simulations available as well and reference it in the paper.

Response:

The computer codes used for simulations and numerical solution of the replicator dynamics are now given in the Supplementary Information, and referenced in the Method section. The data and computer codes are now publicly available at https://doi.org/10.5281/zenodo.4294725, and are referenced in the manuscript.

Reviewer N. 3:

Supplementary Information.

In the later parts of the Results section the author discusses some further results that have been produced, for example in the case of n=3. These results are currently on the Supplementary Information. I believe that the plots accompanying the results should be included in the Results section.

Response:

This is done in the revised manuscript. A subsection and three figures are added (edited and brought to the main text from the Supplementary Information) to discuss the case of $n = 3$ games.

Reviewer N. 3:

Literature.

The author discusses previous literature on some mechanisms of the emergence of cooperation, such as reputation effects, network and spatial structure, voluntary participation, reward, and possibly punishment. However, they do not include discussion on using groups or/and different games. I think that a sentence is missing to clarify where this paper fits in the literature.

Response:

A sentence is added to the introduction which makes connection on some previous work on competition between games. As for using different groups or/and different games, I am not aware of any theoretical study with such an approach and conceptual alignment with this study. However, a discussion regarding the empirical cases where different games can be played is added to the discussion. Connection with voluntary participation is also discussed explicitly in the discussion.

Reviewer N. 3:

Minor and other issues.

- Line 2: the author refers to Publics good game with a capital P, whereas in the rest of the text the author uses a lowercase p.

- Line 49: as shown in the method section. Reference the method section properly.

- Line 52: that the dynamics can settle missing the word that.

- The misuse of commas. There are a few points where the comma is not needed. For example: - Line 38: are formed at random (remove comma) from the population pool. - Line 40: they receive a based payoff pi_0 (remove comma)

- The misuse/overuse of the word here. In several parts of the manuscript the word here can be removed or replaced. For example, following Eq 2, in line 181, line 55, line 146 and line 147.

Response:

I thank the reviewer for bringing my attention to these points. Method section is referenced properly in the revised manuscript, and these corrections are applied. The manuscript is reviewed and edited carefully to remove typos, and to improve the language.
