# Supplementary material for: Freedom to choose between public resources promotes cooperation
Source: PLoS Comput Biol. 2021 Feb 8;17(2):e1008703. doi: 10.1371/journal.pcbi.1008703 (PMC7895419; doi:10.1371/journal.pcbi.1008703)
Supplement: Attachment — Submitted filename: response2.pdf [file pcbi.1008703.s003.pdf]

Responses to review comments

I am thankful to all the reviewers and the editors for careful reading of the manuscript. In the following, I provide my point by point responses to the review comments.

Response to Guest editor's suggestions

Guest editor:
The manuscript has been considerably revised. Now all of the previously consulted reviewers suggest publication of the manuscript, subject to minor adjustments. This is also what I would like to suggest. Please take all the remaining reviewer comments into account when preparing the final version of the manuscript. In addition, please make the following (minor) changes:
Response:
I would like to thank the guest editor once again for the careful reading of my manuscript and many constructive suggestions.
Guest editor:
(-) I appreciate that the author changed the title of the paper, as I believe it would be misleading to speak of "competing public resources". After all, the resources themselves have no strategic interest in attracting individuals; they are not participating in a competition. However, even in the revised manuscript, references to competing resources are found throughout the text. Please revisit each of them and consider revising the respective formulation. For example, the last sentence of the abstract currently reads: "... they can perform the most competitively to attract individuals." This could be rewritten as "they are almost interchangeable" or "they approximately attract the same number of individuals." Alternatively, the sentence could just be omitted.
Response:
I have revisited the references to competition between resources and rephrased them.
Guest editor:
(-) At several places, the author chooses the formulation "Cooperation level is maximized...". This formulation sounds odd to me. Please either use "Cooperation is maximized" or "The cooperation level of the population is maximized"
(-) Page 2, Line 9: "empirical evidence suggests from bacteria to animals and humans..." $->$ "empirical evidence from bacteria to animals and humans suggests..."
(-) Page 2, Line 15: Because most readers (me included) will not know what the difference between "reward" and "reward dilemma" is, please consider combining these two items as "reward"
(-) Page 2, Line 42: "and gather payoff" $->$ "and derive payoffs"
(-) Page 5, Line 146: "As mentioned in the The Model, the model..." $->$ "As mentioned in the section The Model, the framework..."
(-) Page 6, Line 187: "In the region where both r1 and r2 are larger [...] it becomes almost obsolete." Please replace "it" by "the third resource"; otherwise the sentence is ambiguous. Same comment for page 7, line 197.
(-) Page 6, "is not too smaller than that of the two higher-quality resources" $->$ "is not too small compared to the enhancement factor of the two higher-quality resources"
(-) Page 7, line 227: "appear to abound in different species" $->$ "appear to be abound in different species"
Response:
All these changes, but the last one, are applied in the revised version. Regarding the last suggestion, I

checked the usage of "abound", and it appears to me that the sentence "appear to abound in different species" is grammatically correct.

Guest editor:

(-) In general, the author seems to have problems with the correct use of commas. I believe it would be useful if he could ask a colleague for help when preparing the final version of the manuscript.

Response:

I have carefully read the manuscript a few more times, and have edited the language, including the usage of commas. I have also used a language editing software to check for the accuracy.

Guest editor:

(-) I would appreciate if already the intro briefly mentions what the main mechanism for cooperation in this manuscript is (When having the choice between multiple public good games, individuals experience smaller effective group sizes. In sufficiently small groups, cooperation is dominant). Otherwise readers will have to wait until the very end (the Discussion) to really understand what is going on.

Response:

This change is also applied.

Response to Reviewer N. 1

Reviewer N. 1:

The author has satisfactorily taken my comments into account when preparing the revised version of the manuscript. Some very minor issues remain: (-) The first sentence of the author summary should be How is selfishness curbed in many biological populations... instead of How in many biological populations selfishness is curbed.... (-) The caption for Fig. 4 mentions green squares. This should be green circles. (-) Section The case of n=3 resources: the first sentence contains an errant the. (-) page 8, last sentence of the second to last paragraph: fate, not faith. (-) page 8, last paragraph: The author mentioned in the response to the reviewer that preliminary simulations strengthen his assumption that population structure can be beneficial in this setup. Maybe it would be good to very briefly note this, or at least provide a short insight why this assumption seems correct.

Response:

I would like to thank the reviewer once again for the careful reading of the manuscript and constructive suggestions. All these changes are applied in the revised version.

Response to Reviewer N. 2

Reviewer N. 2:

The authors have revised their manuscript comprehensively and with love to detail. I warmly recommend publication in present form.

Response:

I would like to thank the reviewer once again for the careful reading of the manuscript and constructive suggestions.

Response to Reviewer N. 3

Reviewer N. 2:

I would like to open this review by saying that in the point-by-point letter the author thanks each reviewer individually by stating: I am thankful to the reviewer for his careful reading and assessment of the manuscript. Gendered language like this should be avoided in the future. The purpose of this is to avoid word choices which may be interpreted as biased, discriminatory or demeaning by implying that one sex or social gender is the norm.

Overall, the author has taken my comments into account when revising the manuscript. The narrative of the paper, the quality of the figures, and the mathematical explanation of the model have improved.

I have some minor issues regarding the discussion which does not read well in its current form. There are some minor language issues, thus I recommend that the author has a final revision before publishing. Some examples, the abbreviation PGG is used though it is not defined, line 252 misses the word that.

Response:

I would like to thank the reviewer once again for the careful reading of the manuscript and constructive suggestions. I also thank the reviewer for his/her helpful remark.

I have edited the text to improve the language and increase clarity. The mentioned typos in the Discussion are now corrected.
